# Endovascular thrombectomy for acute stroke in anticoagulated patients: systematic review and Meta-Analysis

**DOI:** 10.1007/s11239-025-03192-1

**Published:** 2025-11-02

**Authors:** Kaho Adachi, Allison Raymundo, Anthony Sanchez, Youssef Soliman, Jason Fernando, Morteza Sadeh, Ankit I. Mehta

**Affiliations:** 1 University of Illinois College of Medicine at Chicago, Chicago, IL 60612 USA; 2 Faculty of Medicine, Assiut University, Assiut, Egypt; 3 Department of Neurosurgery, University of Illinois at Chicago, Chicago, IL USA

## Abstract

Endovascular thrombectomy (EVT) is an effective treatment for acute ischemic stroke (AIS). Oral anticoagulants are used for stroke prevention in pro-embolic patients, but their impact on EVT outcomes, particularly symptomatic intracranial hemorrhage (sICH), remains uncertain. This study aims to evaluate the safety and efficacy of vitamin K antagonists (VKAs) and direct oral anticoagulants (DOACs) on EVT outcomes. We searched PubMed, Cochrane, and Embase from 2015 to August 2024 for studies comparing anticoagulated and non-anticoagulated AIS patients undergoing EVT. Safety outcomes included sICH and 90 days mortality. Efficacy outcomes included successful reperfusion rate and 90 days functional outcome. Subgroup analyses evaluated the effect of therapeutic-dose anticoagulation. Fifteen studies with 62,328 AIS patients were included; 9,977 were anticoagulated (6,879 VKA, 3,098 DOAC). VKA patients had a significantly higher rate of sICH (OR = 1.32, 95% CI [1.05, 1.66]) and 90 days mortality (OR = 1.61, 95% CI [1.25, 2.08]) compared to non-anticoagulated patients. DOACs showed no significant difference in sICH risk (OR = 0.83, 95% CI [0.48, 1.44]) or mortality (OR = 1.20, 95% CI [0.89, 1.61]). Functional outcomes at 90 days were significantly worse in both anticoagulated groups, but only VKA patients demonstrated worsened outcomes in the therapeutic-dose subgroup analysis. EVT success rates were comparable between all groups. DOACs offer a safer EVT profile than VKAs, with lower sICH risk and mortality. These findings support DOACs as the preferred anticoagulant for stroke prevention. Further research should assess long-term outcomes and distinguish procedure-related mortality from secondary causes.

**Graphical abstract:**

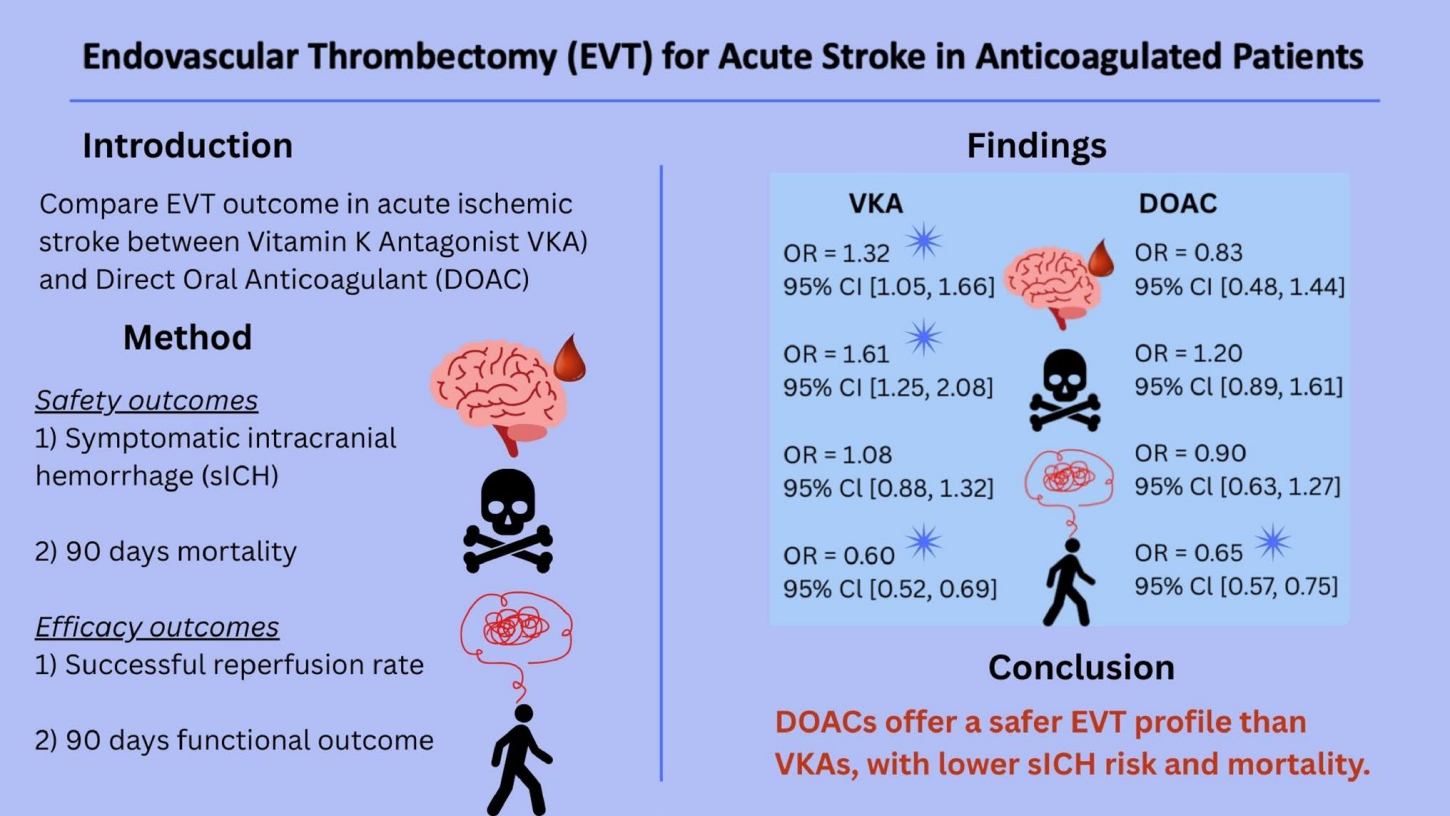

**Supplementary Information:**

The online version contains supplementary material available at 10.1007/s11239-025-03192-1.

## Introduction

Stroke is the fourth highest cause of death in the United States, often resulting from pro-embolic states, including atrial fibrillation (AF), sickle cell anemia, and hypercoagulability disorders [1, 2]. These conditions require prophylactic anticoagulant therapy [2]. Intravenous thrombolysis (IVT) with recombinant tissue-type plasminogen activator has been essential to acute ischemic stroke (AIS) therapy for decades [3]. However, recent studies have shown higher rates of recanalization and improved functional outcomes in AIS patients with endovascular thrombectomy (EVT) when compared to IVT [4]. IVT affects broadly rather than the localized clotted region and is now contraindicated in anticoagulated patients with AIS [5, 6].

The current gold standard therapy for AIS in anticoagulated patients is EVT [7]. However, symptomatic intracranial hemorrhage (sICH) remains a primary concern. AIS patients who are under prophylactic anticoagulant therapy may be at higher risk [8–10]. In the meta-analysis of individual patient data from five randomized controlled trials, the overall risk of sICH in patients receiving EVT was 4.4%. However, the study did not report anticoagulation status or perform subgroup analysis based on the type of anticoagulation [11]. Although there are many studies regarding EVT for AIS in anticoagulated patents, the current literature remains inconclusive. One study found no sICH incidence after EVT in patients receiving vitamin K antagonists (VKAs) or direct oral anticoagulants (DOACs) [12]. Another study, however, found higher rates of sICH in patients receiving VKAs compared with DOACs [10].

A previous meta-analysis assessing the safety and efficacy of EVT in AIS patients receiving OACs showed DOACs as the preferred anticoagulant agent over VKAs [13]. However, newer studies have yielded contradictory results [12, 14–17]. The previous meta-analysis also included patients who only underwent IVT and a control group that included individuals with subtherapeutic anticoagulation rather than exclusively those without anticoagulation, increasing study heterogeneity [13]. These limitations warrant an updated meta-analysis. To address the concerns, this study evaluates the safety and efficacy of EVT in anticoagulated patients, specifically those receiving VKAs and DOACs.

## Method

### Guidelines and search strategy

This review was performed using the Preferred Reporting Items for Systematic Review and Meta-Analysis (PRISMA) guidelines- registration ID: CRD42024598081. We searched the electronic record using PubMed, Embase, and Cochran from 2015 to August 19, 2024, using the keywords (endovascular thrombectomy OR mechanical thrombectomy) AND (anticoagulation OR anticoagulated OR anticoagulant) AND (ischemic stroke).

Inclusion criteria were as follows: Studies comparing anticoagulated and non-anticoagulated patients with ischemic stroke who underwent endovascular or mechanical thrombectomy. Studies that reported outcomes of interest, including sICH, mortality, functional outcome, and reperfusion rate. Types of studies included randomized controlled trials, retrospective and prospective cohort studies, and studies written in English.

Exclusion criteria were as follows: Studies classified as systematic reviews or meta-analyses, case reports, case series, abstracts, or clinical trial registrations. Studies with inconsistent or inadequate data, interventions other than endovascular or mechanical thrombectomy, comparisons other than anticoagulated and non-anticoagulated patients, and studies with diseases other than ischemic stroke were excluded.

## Screening and data extraction

Articles identified by the search engine were exported to Rayyan and used to perform screening. Two reviewers (A.S and A.R) independently conducted literature screening using the title and abstract. Any disagreements were resolved by a third reviewer (K.A). A full-text screening was performed to finalize the list of articles. Two reviewers (A.S and A.R) divided the data extraction work, with a third reviewer (K.A) checking for accuracy. Discrepancies were resolved through discussion. Data was recorded in an Excel spreadsheet.

## Risk of bias

The risk of bias was assessed using the Cochrane Handbook for Systematic Reviews of Interventions under the risk of bias in non-randomized studies of interventions (ROBINS-I). The variables assessed for the non-randomized studies are as follows: bias due to confounding, bias in the selection of participants into the study, bias in classification of interventions, bias due to deviation from intended interventions, bias due to missing data, bias in the measurement of the outcome, and bias in the selection of the reported result. The overall risk of bias was then reported as either low, moderate, or serious.

### Statistical analysis

Random effects models were used to evaluate the safety and efficacy of endovascular or mechanical thrombectomy in anticoagulated compared to non-anticoagulated patients. The Mantel-Haenszel algorithm was used, and the odd ratio (OR) with a confidence interval of 95% was assessed. Subgroup analyses were performed on patients with therapeutic anticoagulants predefined by each studies with available data: INR greater than 1.7 except for one study with INR greater than 2.0; DOAC greater than 50 ng/ml. To assess heterogeneity, the I^2^ statistic was used to quantify heterogeneity. I^2^ = 50% with a p-value < 0.1 was considered indicative of statistically significant heterogeneity. Forest plots were created for each outcome. All were performed using RevMan 5.4 software. In the presence of significant heterogeneity, we conducted a sensitivity analysis using RStudio (Version 4.2.2) for Microsoft Windows to identify the source. This analysis involved sequentially removing individual studies to determine which one introduced heterogeneity into the outcome.

## Result

**Fig. 1:**
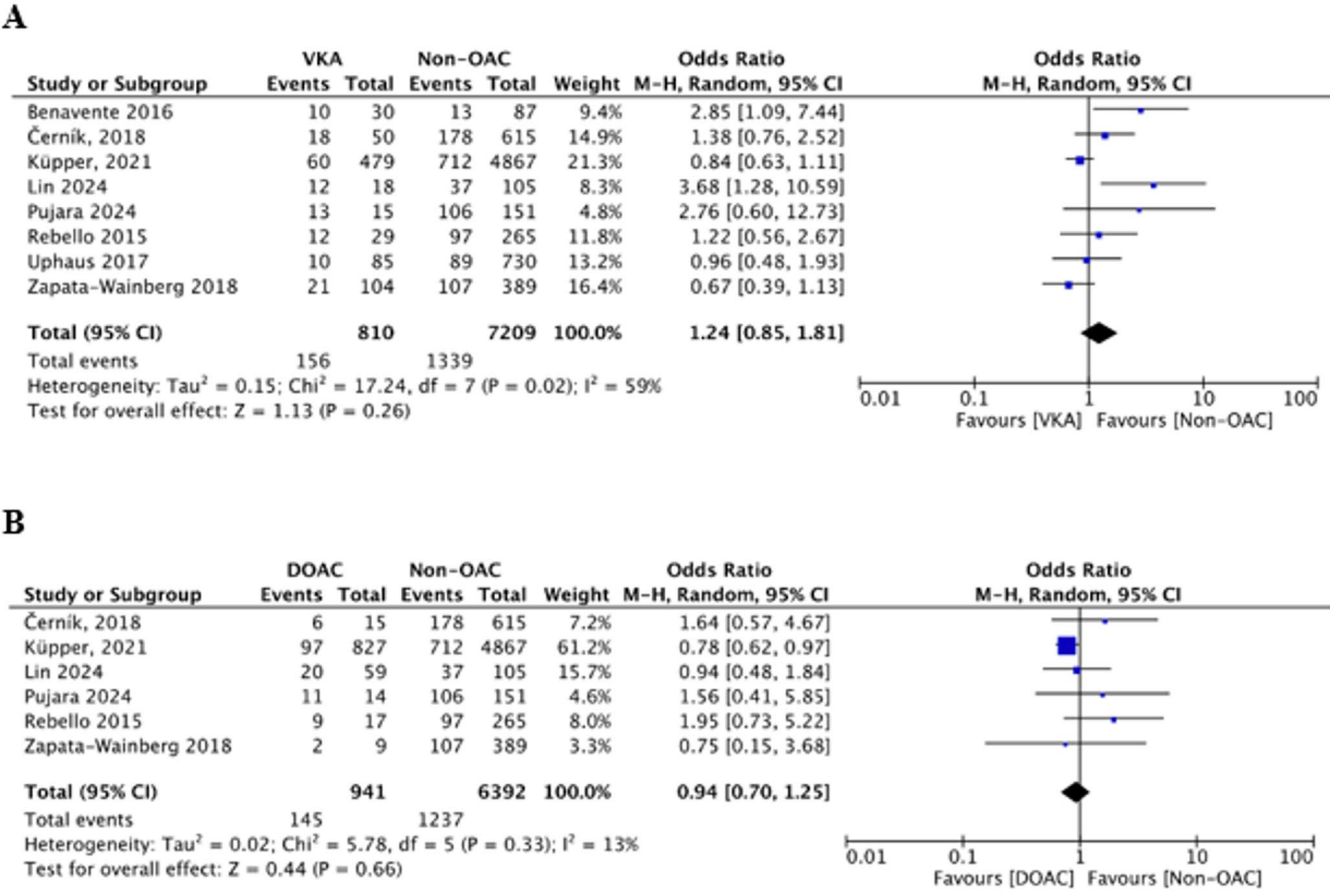
(A) VKA vs. Non-OAC ICH, (B) DOAC vs. Non-OAC ICH

We identified 1177 potentially relevant records, and 172 studies were removed due to duplicates. A total of 953 articles were excluded based on the inclusion and exclusion criteria. 52 articles underwent further screening. 22 records did not compare anticoagulant and non-anticoagulant patients, 6 records were abstract only, 4 records were reviews, 2 records did not have EVT as an intervention, 2 records did not compare VKAs and DOACs, and 1 record did not have enough information on outcomes. Ultimately, 15 studies were included in the final analysis (Supplementary Document Fig. 1) [8–10, 12, 14–24].

### Risk of bias of included studies

**Fig. 2:**
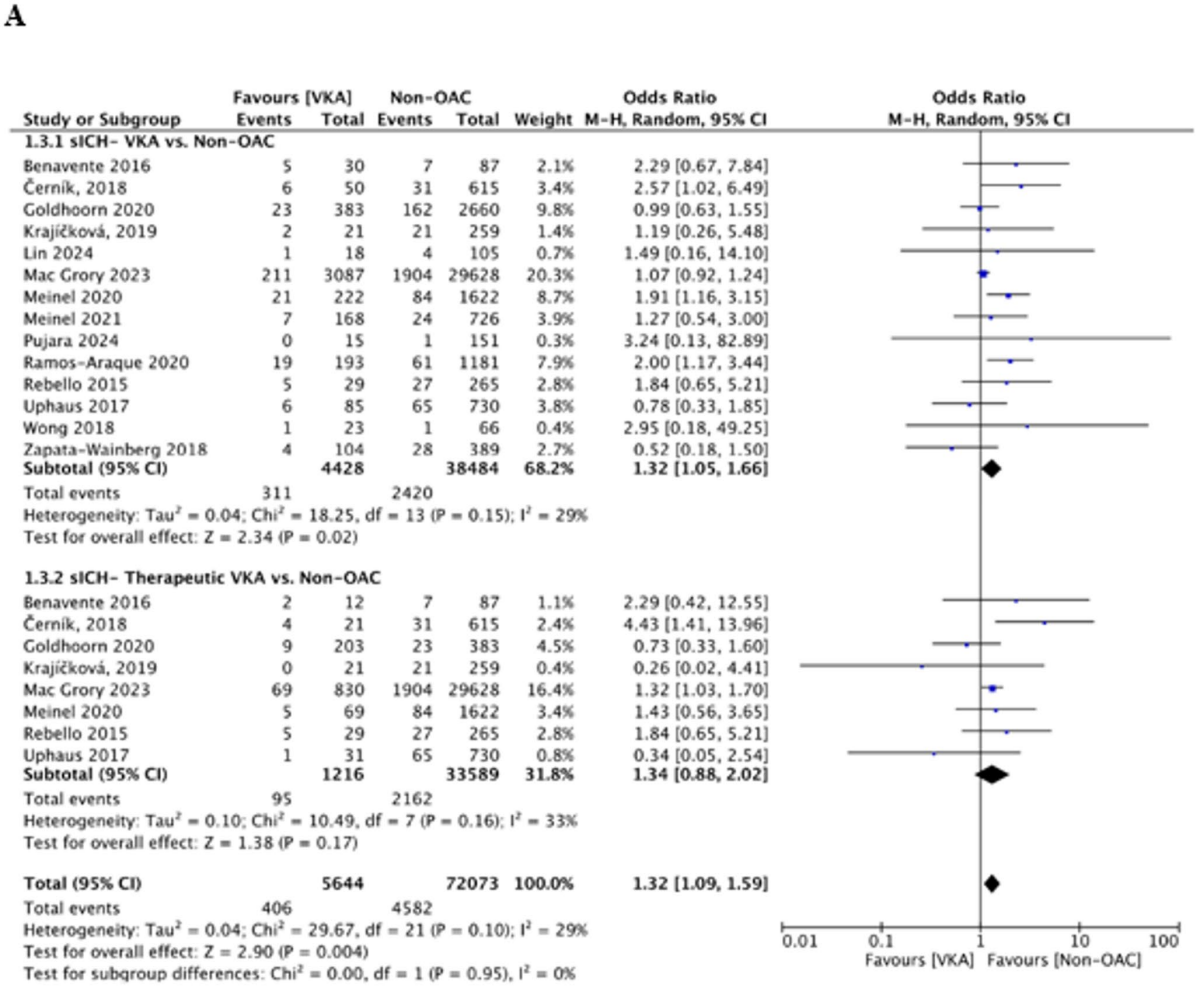

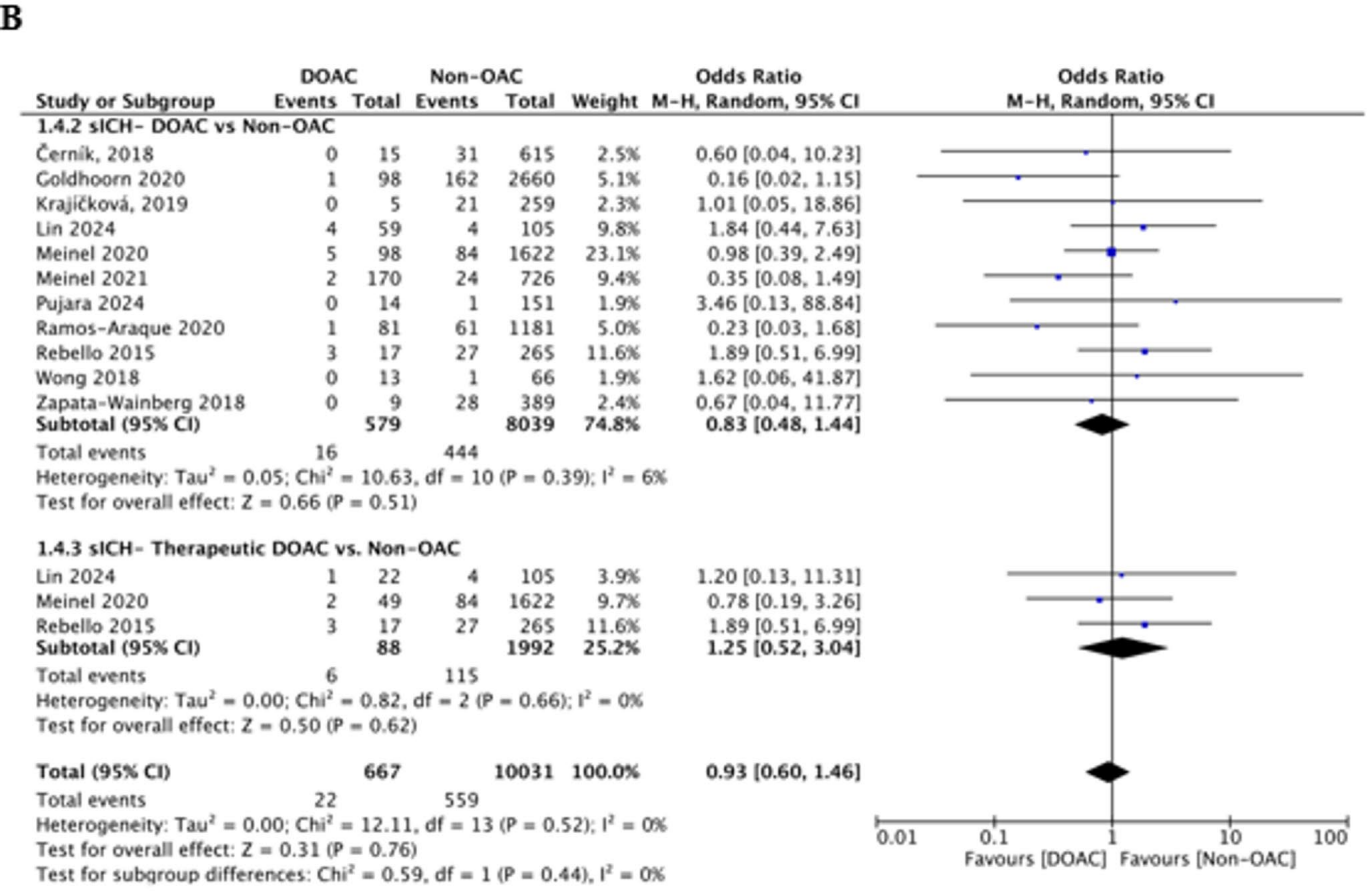
(A) VKA vs. Non-OAC sICH (top); Therapeutic VKA vs. Non-OAC sICH (bottom, (B) DOAC vs. Non-OAC sICH (top); Therapeutic DOAC vs. Non-OAC sICH (bottom)

The risk of bias assessment, according to the Cochrane ROBINS-I, is summarized in Supplementary Document Fig. 2. There were two studies with a low risk of bias, eleven with a moderate risk of bias, and two with a serious risk of bias.

## Study and patient population characteristics

The study and patient characteristics are summarized in the Supplementary Document Tables 1 and 2. There were nine retrospective and six prospective cohort studies. The initial sample size among the fifteen studies included 62,328 patients, with 9,977 OAC patients and 52,351 non-OAC patients. There were 6,879 patients on VKAs and 3,098 patients on DOACs. The mean age ranged from 64 to 79.4 years old in the non-OAC group and 68.6 to 81.3 years old in the OAC group. The mean follow-up was 90 days for both groups, and the main findings are summarized in Supplementary Document Table 1.

## Safety outcomes

**Fig. 3:**
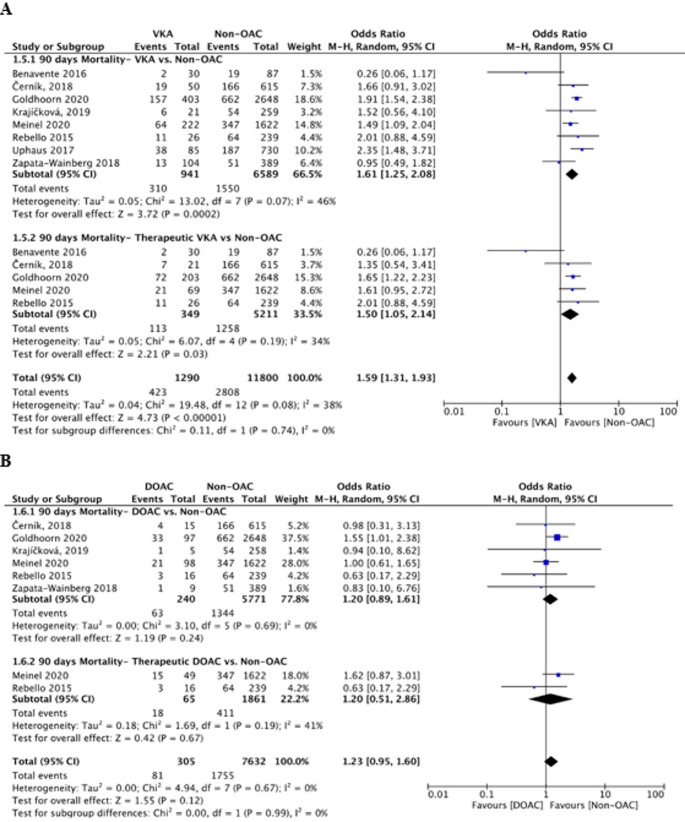
(A) VKA vs. Non-OAC 90 days Mortality (top); Therapeutic VKA vs. Non-OAC 90 days Mortality (bottom), (B) DOAC vs. Non-OAC 90 days Mortality (top); Therapeutic DOAC vs. Non-OAC 90 days Mortality (bottom)

The ICH results are summarized in Fig. 1. The overall ICH rate between VKA and non-OAC saw no significant difference (OR = 1.24, 95% CI [0.85, 1.81]). Sensitivity analysis confirmed that heterogeneity remained greater than 50% after the sequential omission of individual studies, and the effect size remained nonsignificant (Supplementary Document Fig. 3). Similarly, the overall ICH rate between DOAC and non-OAC was not significant (OR = 0.94, 95% CI [0.70, 1.25]). For sICH, VKA was associated with a significantly higher rate compared to non-OAC (OR = 1.32, 95% CI [1.05, 1.66]). However, in the subgroup analysis, no difference was observed between therapeutic-dose VKA and non-OAC (OR = 1.34, 95% CI [0.88, 2.02]) (Fig. 2A). sICH rates for DOAC and therapeutic-dose DOAC also revealed no significant differences compared to non-OAC (OR = 0.83, 95% CI [0.48, 1.44] and OR = 1.25, 95% CI [0.52, 3.04], respectively) (Fig. 2B). 90 days mortality showed a significantly increased rate in VKA compared to non-OAC (OR = 1.61, 95% Cl [1.25, 2.08]). The subgroup analysis result comparing therapeutic-dose VKA to non-OAC remained significant (OR = 1.50, 95% Cl [1.05, 2.14]) (Fig. 3A). However, no difference was seen between DOAC or therapeutic DOAC compared to non-OAC (OR = 1.20, 95% Cl [0.89, 1.61] and OR = 1.20, 95% Cl [0.51, 2.86], respectively) (Fig. 3B).

### Efficacy outcomes

**Fig. 4:**
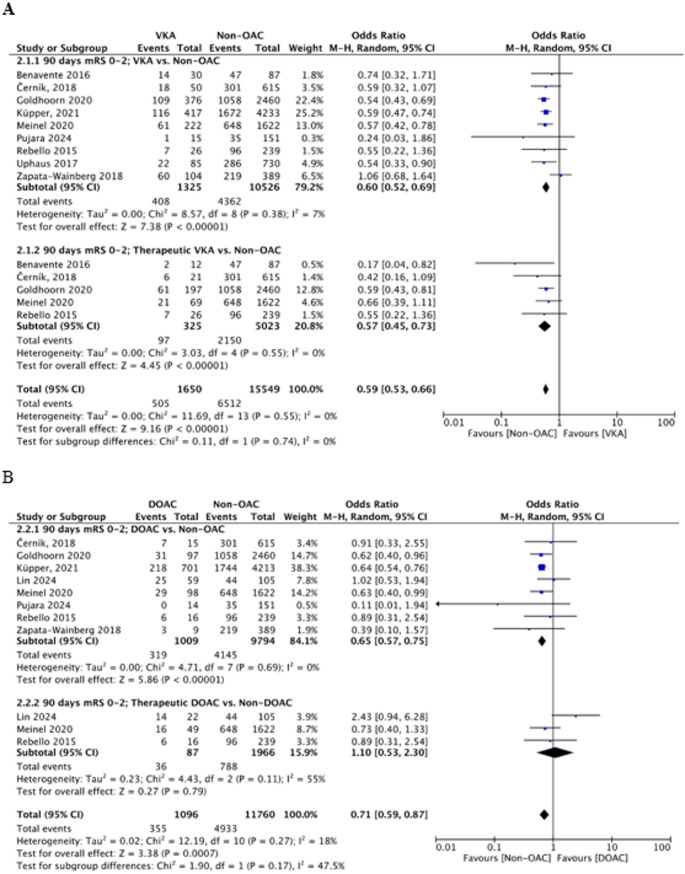
(A) VKA vs. Non-OAC 90 days mRS 0–2 (top); Therapeutic VKA vs. Non-OAC 90 days mRS 0–2 (bottom), (B) DOAC vs. Non-OAC 90 days mRS 0–2 (top); Therapeutic DOAC vs. Non-OAC 90 days mRS 0–2 (bottom)

**Fig. 5:**
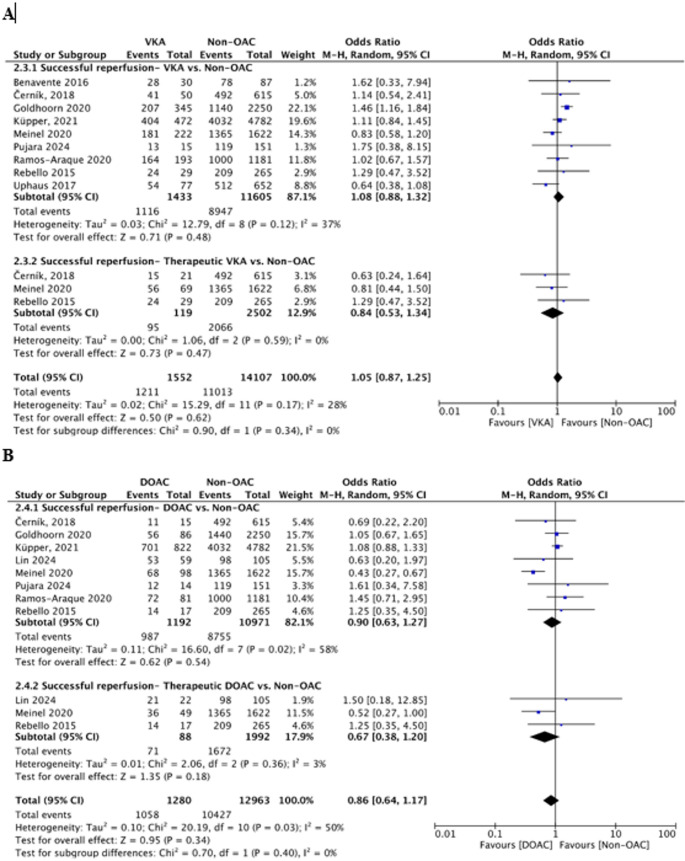
(A) VKA vs. Non-OAC successful reperfusion (top); Therapeutic VKA vs. Non-OAC successful reperfusion (bottom), (B) DOAC vs. Non-OAC successful reperfusion (top); Therapeutic DOAC vs. Non-OAC successful reperfusion (bottom)

90 days Modified Rankin Scale (mRS) results are summarized in Fig. 4. Our study showed that VKA had significantly worse functional outcomes than non-OAC (OR = 0.60, 95% Cl [0.52, 0.69]). The result of subgroup analysis with therapeutic-dose VKA remained the same (OR = 0.57, 95% Cl [0.45, 0.73]). 90 days mRS in DOAC also demonstrated worse functional outcomes compared to non-OAC (OR = 0.65, 95% Cl [0.57, 0.75]). The result became nonsignificant in the subgroup analysis with therapeutic-dose DOAC (OR = 1.10, 95% Cl [0.53, 2.30]). Sensitivity analysis for the therapeutic-dose DOAC showed that omitting two of the studies independently showed heterogeneity of less than 50%. However, the result remained nonsignificant (Supplementary Document Fig. 4). Successful reperfusion was nonsignificant for both VKA vs. non-OAC and in the subgroup analysis of therapeutic-dose VKA vs. non-OAC (OR = 1.08, 95% Cl [0.88, 1.32] and OR = 0.84, 95% Cl [0.53, 1.34], respectively) (Fig. 5 A). Successful reperfusion in DOAC also showed nonsignificance for both DOAC vs. non-OAC and therapeutic-dose DOAC vs. non-OAC (OR = 0.90, 95% Cl [0.63, 1.27] and OR = 0.67, 95% Cl [0.38, 1.20], respectively) (Fig. 5B). Sensitivity analysis for DOAC vs. non-OAC showed heterogeneity of 0% after omitting one study, and the result remained nonsignificant after an individual study was omitted (Supplementary Document Fig. 5).

### Meta analyses comparison

A side-by-side comparison with the previous meta-analysis by Chen et al. (2022) is presented in Supplementary Document Table 3. Both studies included 15 studies; however, our analysis incorporated 5 additional recent studies not included in the earlier work. Unlike Chen et al., our study restricted inclusion to patients who underwent EVT and consistently compared outcomes between anticoagulated and non-anticoagulated patients. Sensitivity analysis was conducted in our study to assess the impact of individual studies on heterogeneity; this was not reported in the previous meta-analysis. Regarding outcomes, both analyses demonstrated that DOACs were associated with lower rates of sICH and mortality and improved functional outcomes. For both studies, no differences were observed in the successful reperfusion rate for VKAs and DOACs.

## Discussion

The purpose of this meta-analysis was to assess the safety and efficacy of EVT in patients taking VKAs or DOACs and compare these groups to non-OAC patients. The study population comprised adults with AIS caused by large vessel occlusion and treated with EVT. Common exclusion criteria across these studies included prior severe disability, fatal malignancy, and anticoagulation with heparin.

### Risk of intracerebral hemorrhage

ICH is a feared complication in stroke management associated with a high mortality rate [25]. The risk of ICH following EVT varies widely with rates of 12% to 40% [26–28]. In this study, VKA was associated with a significantly higher rate of sICH, regardless of therapeutic levels. Despite including four times the larger sample size, newer studies, and minimizing confounding variables, the findings were consistent with the previous meta-analysis [12, 13]. One study showed that VKA was an independent predictor of sICH in AIS patients undergoing EVT [9]. Additionally, compared to DOACs, VKAs have a longer half-life and require more time to reverse in the event of hemorrhage. This delayed reversal can lead to larger hematoma volumes and worse clinical outcomes [29]. Long-term VKA use is also associated with vascular calcification and endothelial dysfunction, which may predispose patients to ICH, even when INR is within the recommended range [30]. Our results may also be explained by the increased rate of AF among VKA patients [15, 20, 23]. AF is a risk factor for cardioembolic stroke, which can lead to hemorrhagic transformation and ICH [8, 31]. Therefore, the increased rate of sICH in VKA may be attributed to the underlying comorbidities rather than the type of anticoagulation. Adequate anticoagulation improves control of AF, and our result shows the nonsignificant difference of sICH in the therapeutic VKA level. Previous studies have shown that lower plasma clot permeability is independently associated with an increased risk of major bleeding [32, 33]. Plasma clots in patients receiving DOACs, such as dabigatran and rivaroxaban, are more permeable, while VKA showed decreased permeability [33–35]. As seen, the underlying clot structure reflects the risk of ICH, with DOAC indicating an increased safety profile.

Three studies, though nonsignificant, showed that VKA was associated with a lower rate of sICH [22, 24, 36]. In one study, the patient population was younger, with a median age of 62, compared to other papers with a median age of 78 and 71 for their OAC and control, respectively [19, 36]. This younger population correlates to an overall healthier vasculature. Another study reported that most of the VKA patients had an INR less than 2, and the study may not be representative of the patient population in the United States [22].

### Successful reperfusion

EVT aims to restore adequate cerebral perfusion through successful recanalization, often measured by the Thrombolysis in the Cerebral Infarction (TICI) grading system, where TICI 2b–3 indicates optimal reperfusion [37]. Possible complications of EVT include vasospasm, vessel perforation, and distal embolization [38, 39]. Theoretically, anticoagulation may improve flow during reperfusion by reducing clot burden and facilitating vessel clearance [40]. However, this meta-analysis demonstrates that anticoagulated patients, whether on VKAs or DOACs, achieve reperfusion outcomes similar to non-anticoagulated patients. This suggests that anticoagulation does not benefit or impair recanalization success. Several studies support these results, reporting that anticoagulated patients achieve TICI 2b–3 scores at similar rates to non-anticoagulated patients [13, 41, 42]. Our finding is crucial for patient care, as successful reperfusion is directly associated with improved neurological recovery and long-term functional outcomes [43].

### 90 days functional outcomes and mortality

Overall, VKAs and DOACs both had worse 90 days functional outcomes compared to non-OAC patients. However, only VKAs had a worse outcome in the therapeutic dosage subgroup analysis. Previous studies found factors such as sICH and successful recanalization independently predict functional outcomes. For anticoagulated patients, while sICH is consistent with our outcomes, successful reperfusion may not be applicable as the rate was similar among VKA, DOAC, and non-OAC patients [44].

90 days mortality is an important indicator of early procedural outcomes and longer-term risks. Our study showed that patients taking VKA had a significantly higher 90 days mortality compared to the control, which is consistent with prior research. This may be explained by the increased rate of sICH observed in VKA patients as sICH has been shown to predict mortality in stroke management [13]. Given the 90-day follow-up period, mortality may not exclusively reflect procedure-related causes. Due to the underlying conditions, anticoagulated patients have inherently higher baseline risk for recurrent cerebrovascular events [31]. This is more concerning for VKA as a recent study showed a higher rate of both ischemic and hemorrhagic stroke in patients taking VKA compared to those taking DOAC [45]. Further research should highlight perioperative and in-hospital mortality to better delineate death directly related to the EVT procedures.

### Vitamin K antagonists versus direct oral anticoagulants

Overall, DOACs demonstrated higher safety and efficacy compared to VKA for patients undergoing EVT. However, other practical measures should be considered. VKAs are more affordable and accessible, particularly in resource-limited settings, but they require regular INR monitoring and adherence to dietary restrictions [46]. This increases the burden on healthcare resources and may complicate long-term adherence​. DOACs, on the other hand, offer several practical advantages, including predictable pharmacokinetics, fewer drug and dietary interactions, and exemption from routine laboratory monitoring, making them ideal for patients with barriers to frequent medical follow-up, complex medication management, or limited healthcare access. However, DOACs are more expensive and may not be suitable for patients with renal impairment or higher bleeding risk [47]. The choice should ultimately balance financial cost, access to healthcare resources, and the risk of requiring future EVT.

### Meta-Analysis comparison

Our study offers a comprehensive analysis while minimizing confounding effects. The previous meta-analysis included a study with patients who underwent only IVT [48]. It also included one study where the control group included patients with subtherapeutic anticoagulation levels rather than no anticoagulation [49]. These factors limit the ability to isolate the effects of anticoagulation on EVT outcomes. The analysis was also limited to sICH, while our study also included all ICH, providing a more comprehensive assessment of EVT risk in anticoagulated patients (Table 1).

### Limitations

The primary limitation of this study is the heterogeneity due to variations in study design and patient selection. Notably, one study excluded patients with an INR greater than 3.5, and another excluded those with an INR below 1.7.^8,21^ Additionally, some studies included only VKAs, limiting insights into DOAC safety in this population. We reported I² values, conducted sensitivity analyses, and provided a detailed baseline characteristics of each included study to address such limitations and offer context for interpreting the findings. Our ability to assess long-term outcomes was restricted, as follow-up was limited to 90 days. Furthermore, the lack of delineation between perioperative, in-hospital, and follow-up mortality limited our ability to distinguish deaths directly related to the procedure from those due to secondary stroke or unrelated causes. Although publication bias is a potential concern in any meta-analysis, the inclusion of many studies reporting no significant difference in primary outcomes (e.g., ICH between anticoagulant and non-anticoagulant groups) suggests a reduced risk of such bias in this analysis. We also limited our analysis to studies published in English, which may introduce selection bias. This could disproportionately reflect outcomes from English-speaking or higher-resource settings. Lastly, future research is warranted to investigate the specific effect of anticoagulation in EVT based on clot compositions, including lipid-rich emboli and cardioembolic clots.

## Conclusion

Our meta-analysis demonstrates that DOACs have a better safety and efficacy profile compared to VKAs. VKAs had a significantly higher rate of sICH, mortality, and poor functional outcomes. Contrarily, DOACs only showed a significantly worse functional outcome, but no difference was seen among patients on therapeutic dosage. Stroke management should increasingly favor the use of DOACs over VKAs in patients who may require EVT, given their safety and efficacy advantages.

## Supplementary Information

Below is the link to the electronic supplementary material.

Supplementary Material 1

## Data Availability

No datasets were generated or analysed during the current study.
