# Supplementary material for: Endovascular thrombectomy for acute stroke in anticoagulated patients: systematic review and Meta-Analysis
Source: J Thromb Thrombolysis. 2025 Nov 2;59(3):773–85. doi: 10.1007/s11239-025-03192-1 (PMC13246563; doi:10.1007/s11239-025-03192-1)
Supplement: Supplementary file 1 — Supplementary Material 1 [file 11239_2025_3192_MOESM1_ESM.docx]

**Supplementary Data**

**Title:** Endovascular Thrombectomy for Acute Stroke in Anticoagulated Patients: Systematic Review and Meta-Analysis

**Authors:** Kaho Adachi, BA^1^, Allison Raymundo, BS^1^, Anthony Sanchez, MS^1^, Jason Fernando, BS^1^, Morteza Sadeh, MD PHD^2^, Ankit I. Mehta, MD, FAANS, FACS^1,2^

**Author Affiliations:**

^1^University of Illinois College of Medicine at Chicago, Chicago, IL 60612

^2^Department of Neurosurgery, University of Illinois at Chicago, Chicago, IL 60612

Corresponding Author’s Email: [ankitm@uic.edu](mailto:ankitm@uic.edu)

Figure 1. PRISMA flow diagram


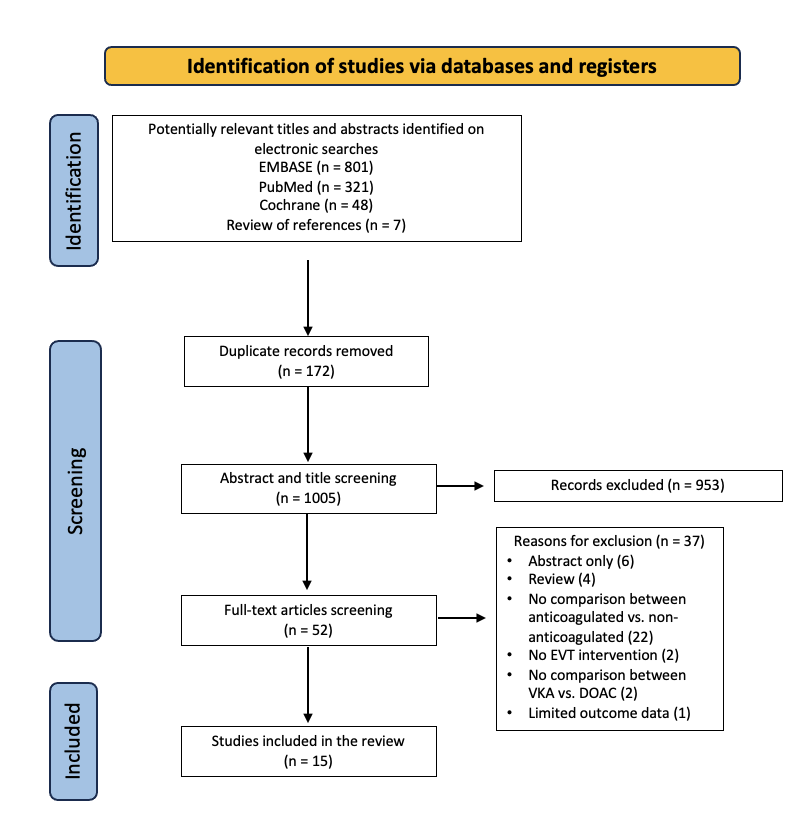


Figure 2. Risk of bias for non-RCTs


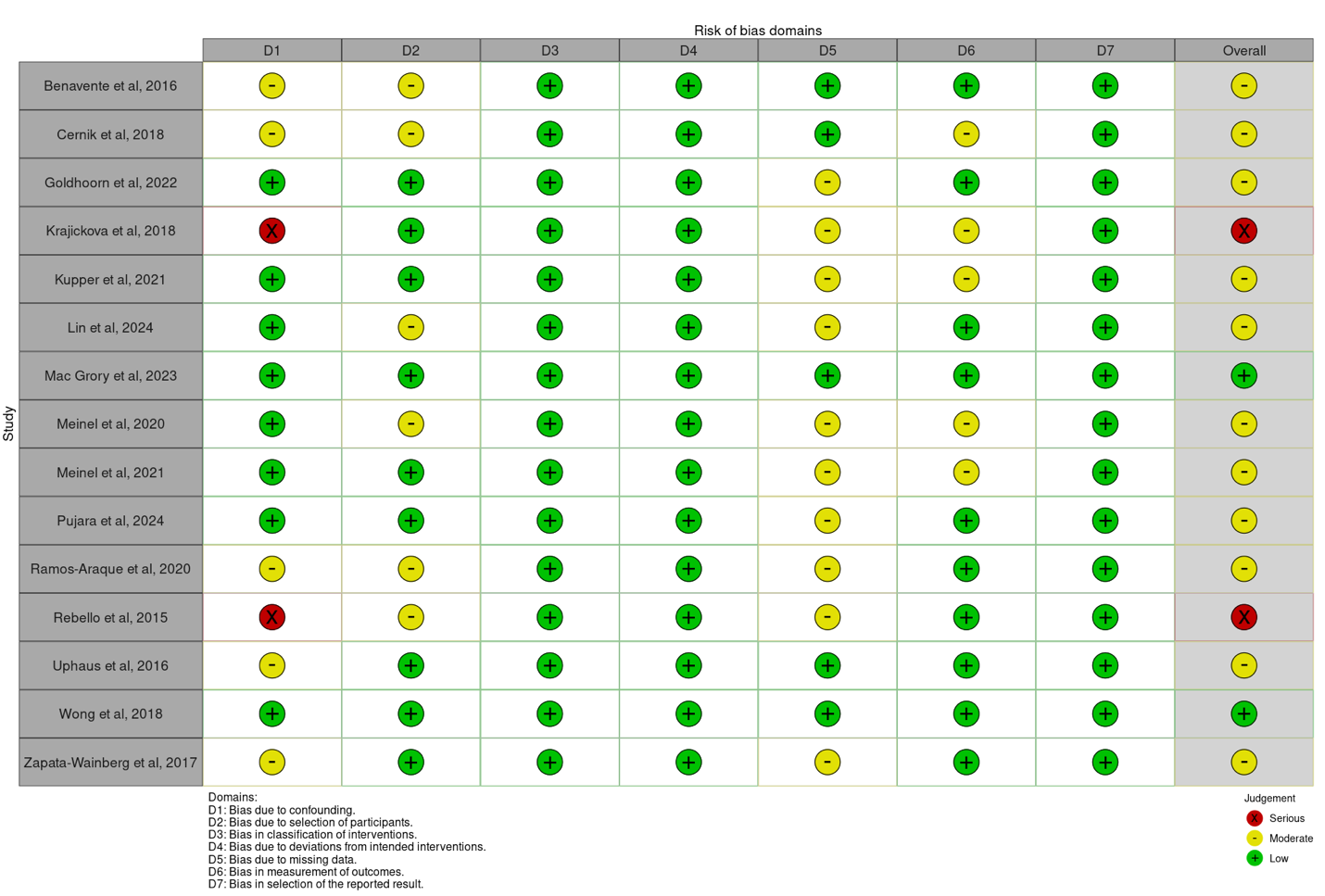


Table 1. Study Characteristics

| First Author, year | Country | Study Design | Study Population | Follow-up (days) | Intervention | Summary/ Main findings |
| --- | --- | --- | --- | --- | --- | --- |
| Benavente, 2016 | Spain | Prospective study | AIS patients treated with EVT | 90 | anticoagulant (VKA) vs no-anticoagulant | None of the outcomes reached statistical significance. Recanalization rates were similar between groups, while functional outcomes (mRS ≤ 2 at 3 months) were slightly lower in anticoagulated patients. sICH was higher in anticoagulated patients, but mortality at 3 months was lower compared to non-anticoagulated patients. |
| Černík, 2018 | Czech Republic | Retrospective study | AIS patients w/ MCA, ICA or Basilar occlusion treated with EVT | 90 | anticoagulant (warfarin, DOAC, & LMWH) vs no-anticoagulant | Anticoagulated patients showed significantly worsened clinical outcomes after 90 days (3+ mRS score). No statistically significant differences were seen between the groups for TICI, ICH/sICH, or mortality at 90 days. |
| Goldhoorn, 2020 | Netherlands | Prospective study | AIS patients treated with EVT (anterior and posterior circulation) | 90 | anticoagulant (VKA and DOAC) vs no-anticoagulant | All reperfusion rates were similar between groups. There was no statistically significant difference in sICH between groups. NIHSS score postintervention and 90 day mortality were both initially higher in anticoagulated group however found to be statistically insignificant after adjustment for prognostic factors. Use of anticoagulation was associated with worse outcomes on the mRS in the unadjusted analysis but also found to be statisically insignficant after prognostic factor adjustment. |
| Krajíčková, 2019 | Czech Republic | Prospective study | Anterior circulation ischemic stroke patients treated with EVT | 90 | anticoagulant (VKA and DOAC) vs no-anticoagulant | No statistically significant differences were observed between groups for rate of successful recanalisation (TICI ≥2b), occurence of ICH or sICH, good 90-day clinical outcome, or 90-day mortality rate. |
| Küpper, 2021 | Germany | Prospective study | Large vessel occlusion stroke patients treated with EVT | 90 | anticoagulant (VKA and NOAC) vs no-anticoagulant | Endovascular treatment efficacy was similar among anticoagulated patients, including VKAs and NOACs, compared to non-OAC patients. However, good functional outcomes at 90 days were less frequent in anticoagulated patients (p < 0.005). Anticoagulation use was not associated with an increased risk of ICH at 24 hours. |
| Lin, 2024 | Taiwan | Prospective study | AIS patients with AFib treated withg EVT | 90 | anticoagulant (VKA and DOAC) vs single or dual antiplatelet vs no-anticoagulant | No significant differences were observed in functional independence at 3 months or return to premorbid status among the groups. Patients with low DOAC levels were less likely to achieve functional independence, while those with therapeutic anticoagulation had better outcomes. sICH rates were slightly higher in low-level DOAC users. Thrombus permeability was similar across groups. |
| Mac Grory, 2023 | USA | Retrospective study | AIS patients due to large vessel occlusion treated with EVT | no follow up data | anticoagulant (VKA) vs no-anticoagulant | No signifiant difference in the rate of sICH was observed. Hwoever, the risk was significantly increased among the subgroup of patients with recent VKA use and an elevated INR compared to control. |
| Meinel, 2020 | Switzerland | Retrospective study | AIS patients treated with EVT | 90 | anticoagulant (VKA and DOAC) vs no-anticoagulant | Patients taking VKA have an increased risk of sICH and mortality compared to control. |
| Meinel, 2021 | Switzerland | Retrospective study | AIS patients with AFib treated with IVT/ EVT | 90 | anticoagulant (VKA and DOAC) vs no-anticoagulant | Stroke severity was significantly lower in DOAC compared to VKA and control. Rate of sICH after MT were similar across groups. DOAC had the highest rate of favorable 3-month functional outcomes, though this difference was not significant. |
| Pujara, 2024 | USA | Retrospective study of RCT | Large core strokes patients treated with EVT | 90 | anticoagulant (VKA and DOAC) vs no-anticoagulant | EVT did not improve outcomes in anticoagulated patients with large core infarcts, unlike in non-anticoagulated patients. The anticoagulated group had higher comorbidities, but results remained consistent after adjustment. Hemorrhage rates were higher with EVT in anticoagulated patients, but no sICH was observed. |
| Ramos-Araque, 2020 | Spain | Retrospective study | AIS patients treated with EVT | 90 | anticoagulant (VKA and DOAC) vs no-anticoagulant | VKA was an independent predictor of sICH after EVT. This excess risk was associated neither with INR value by the time thrombectomy was performed, nor with a worse functional outcome or mortality at 3 months. |
| Rebello, 2015 | USA | Retrospective Study | Large vessel occlusion stroke patients treated with EVT | 90 | anticoagulant (VKA and NOAC) vs no-anticoag | There were no statistically significant differences in the rates of parenchymal hematoma, 90-day modified Rankin Scale score of 0 to 2, and 90-day mortality among anticoagulated and control patients. Similarly, there were no significant differences between anticoagulated and tPA patients regarding parenchymal hematoma, 90-day modified Rankin Scale score of 0 to 2, and 90-day mortality. |
| Uphaus, 2017 | Germany and Austria | Prospective study | AIS patients treated with EVT | 90 | anticoagulant (VKA) vs no-anticoagulant | VKA use did not significantly impact clinical outcomes or ICH rates after EVT. After adjusting for confounders, anticoagulation status was not associated with worse outcomes. |
| Wong, 2018 | Australia | Retrospective study | Anterior or posterior large vessel occlusion patients treated with EVT (did not receive IV tPA or intra-arterial thrombolysis) | 90 | anticoagulant (VKA and DOAC) vs no-anticoagulant | There were no significant differences in ICH in anticoagulated patients compared to non-anticoagulated patients. No cases of sICH were observed among patients taking DOACs. After 90 days of follow-up, the rates of functional independence and mortality were also similar between the two groups. |
| Zapata-Wainberg, 2018 | Spain | Retrospective study | AIS patients treated with EVT | 90 | anticoagulant (VKA and DOAC) vs no-anticoagulant | Rates of ICH and recanalization after treatment were similar between non-anticoagulated and anticoagulated patients. After 3 months of follow-up, mRS scores and mortality rate were similar between the two groups. No differences were found for ICH, mRS score, or mortality rates between VKA and DOAC. |

**Abbreviations**: AIS, Acute Ischemic Stroke; EVT, Endovascular Thrombectomy; VKA, Vitamin K Antagonist; DOAC, direct oral anticoagulant; NOAC, Novel Oral Anticoagulant; LMWH, Low Molecular Weight Heparin; mRS, modified Rankin Scale; sICH, symptomatic Intracranial Hemorrhage; TICI, Thrombolysis in Cerebral Infarction; AFib, Atrial Fibrillation; RCT, Randomized Controlled Trial; IVT, Intravenous Thrombolysis; tPA, tissue Plasminogen Activator.

Table 2. Baseline Characteristics of Patient Population

| **First Author, year** | **Group** | **Sample Size** | **Age** Median (IQR) Mean ± SD | **Sex, Male** n (%) | **A-fib** n (%) | **Site of vessel occlusion** n (%) | **IV tPA administration** n (%) | **NIHSS** Median (IQR) Mean ± SD | **ASPECT score** n (%) Median (IQR) Mean ± SD | **Onset to hospital time/ Time from onset of sxs to recanalization** Median (IQR) Mean ± SD | **Final score on TICI** n (%) |
| --- | --- | --- | --- | --- | --- | --- | --- | --- | --- | --- | --- |
| Benavente, 2016 | VKA | 30 | 72.8 ± 7.85 | 18 (60) | (87.49) | M1: (60) M2: (4.17) TICA: (29.17) tandem ICA-IC: (4.17) AB +/- P1: (12.51) Cervical ICA: (0.0) Tandem EC-IC + Cervical ICA: (0.0) | NR | 17 (7-28) | NR | NR | NR |
|  | Non-OAC | 87 | 67.07 ± 10.60 | 55 (63.21) | (17.44) | M1: (45.16) M2: (9.68) TICA: (16.13) Tandem ICA-IC: (17.74) AB +/- P1: (6.44) Cervical ICA: (4.84)  Tandem EC-IC + Cervical ICA: (19.35) | NR | 16 (2-24) | NR | NR | NR |
| Černík, 2018 | VKA | 50 | 76 ± 11 |  |  |  |  |  |  |  |  |
|  | DOAC | 15 | 77 ± 6 |  |  |  |  |  |  |  |  |
|  | Non-OAC | 615 | 70.0 ± 12.5 | 314 (51) | 230 (38) | MCA: 503 (82) ICA: 145 (24) BA: 76 (12) | NR | 17 (1-42) | NR | 240 ± 98 | (TICI ≥2b) 492 (80) (TICI 3) 394 (64) |
|  | OAC | 88 | 75.5 ± 11.8 | 40 (46) | 74 (84) | MCA: 73 (83) ICA: 16 (18) BA: 12 (14) | NR | 16.5 (2-36) | NR | 225 ± 73 | (TICI ≥ 2b) 70, (80) (TICI 3) 57, (65) |
| Goldhoorn, 2020 | OAC | 502 | 78 (69-84) | 262 (52) | 394 (78) | ICA (intracranial): 12 (2) ICA-T 109: (22) M1: 280 (56) M2: 72 (14) M3 or ACA: 3 (1) | 173 (34) | 17 (12-20) | 0-4: 14 (3) 5-7: 87 (17)  8-10: 381 (76) | NR | (TICI ≥ 2b) 264 (61) (TICI ≥ 2c): 187 (43) (TICI 3) 134 (31) |
|  | Non-OAC | 2,660 | 71 (60-80) | 1384 (52) | 359 (13) | ICA (intracranial): 143 (5) ICA-T: 525 (20) M1: 1,476 (55) M2: 366 (14) M3 or ACA: 21 (1) | 2,239 (84) | 16 (11-19) | 0-4: 129 (5) 5-7: 540 (20) 8-10: 1,906 (72) | NR | (TICI ≥ 2b) 1440 (64) (TICI ≥ 2c) 979 (43) (TICI 3) 709 (31) |
| Krajíčková, 2019 | OAC | 26 | 75±8.0 | 10 (38.5) | 26 (100) | MCA-M1: 18 (69.2) MCA-M2: 2 (11.5) Distal ICA + M1/A1: 5 (19.2) Tandem: 0 (0.0) | NR | 15 (1-28) | NR | NR | (TICI ≥2b) 18 (69.2) |
|  | Non-OAC | 259 | 71.1±13.8 | 105 (40.5) | 114 (44) | MCA-M1: 145 (56) MCA-M2: 33 (12.7) Distal ICA + M1/A1: 38 (14.7) Tandem: 43 (16.6) | NR | 14 (0-40) | NR | NR | (TICI ≥ 2b) 214 (82.6) |
| Küpper, 2021 | VKA | 479 | 77.7±10.9 | 227 (47.4) | 413 (86.9) | ECA: 22 (4.6) ICA w/o Carotid-T: 26 (5.5) ICA w/ Carotid-T: 91 (19.1) M1 proximal: 142 (29.8) M1 distal: 93 (19.5) M2: 100 (21.0) ACA: 18 (3.8) PCA: 14 (2.9) Basilar: 35 (7.4)  Vertebral: 9 (1.0) | 128 (26.7) | 15 (0-42) | 9 (1-10) | NR | (mTICI 2b–3) 404, 85.6% |
|  | NOAC | 827 | 77.7±10.9 | 358 (42.2) | 715 (87.5) | ECA: 21 (2.6) ICA w/o Carotid-T: 30 (3.7) ICA w/ Carotid-T: 135 (16.5) M1 proximal: 280 (34.3) M1 distal: 161 (19.7) M2: 172 (21.1) ACA: 22 (2.7) PCA: 26 (3.2) Basilar: 71 (8.7)  Vertebral: 5 (1.1) | 91 (11.0) | 15 (0-42) | 9 (1-10) | NR | (mTICI 2b–3) 701 (85.3) |
|  | Non-OAC | 4,867 | 72.0±13.5 | 2,458 (50.5) | 1,438 (29.9) | ECA: 333 (6.9) ICAw/o Carotid-T: 261 (5.4) ICA w/ Carotid-T: 746 (15.5) M1 proximal: 1,629 (33.9) M1 distal: 977 (20.3) M2: 982 (20.5) ACA: 104 (2.2) PCA: 122 (2.5) Basilar: 485 (10.1)  Vertebral: 96 (2.0) | 2,892 (59.4) | 14 (0-42) | 9 (1-10) | NR | (mTICI 2b–3) 4032 (84.3) |
| Lin, 2024 | OAC | 85 | 75.8 ± 9.9 | 36 (42.4) | 3 (3.5) | NR | 0 (0) | 18 (14-23) | 8 (7-10) | NR | (mTICI 2b–3) 76 (89.4) |
|  | Non-OAC | 105 | 75.5 ± 11.3 | 51 (48.6) | 56 (53.3) | NR | 2 (1.9) | 18 (14-22) | 8 (7-10) | NR | (mTICI 2b–3) 98 (93.3) |
| Mac Grory, 2023 | VKA | 3,087 | 74.88 | 1,432 (46.4) | 2,530 (82.0) | MCA: 1,762 (86.5) ICA: 357 (17.5) Other cerebral artery branch: 73 (3.6) Basilar: 56 (2.7) Vertebral: 10 (0.5) | 1,313 (42.53) | 18 (14-23) | N/A | 209 (154-265) | NR |
|  | Non-OAC | 29,628 | 74.88 | 14,691 (49.6) | 7,561 (25.5) | MCA: 17,508 (84.7) ICA: 3,936 (19.0) Other cerebral artery branch: 760 (3.7) Basilar: 812 (3.9) Vertebral: 248 (1.2) | 23,290 (78.61) | 17 (13-22) | N/A | 205 (152-268) | NR |
| Meinel, 2020 | DOAC | 318 | 78 (70-83) | 49 (50) | NR | NR | 7 (7.1) | 16 (8.5-19.5) | 9 (7-10) | NR | NR |
|  | VKA | 855 | 79 (71-84) | 103 (46.4) | NR | NR | 29 (17.6) | 16 (11-20) | 9 (7-10) | NR | NR |
|  | Non-OAC | 6,289 | 73 (60-81) | 826 (50.9) | NR | NR | 39 (17.6) | 16 (10-20) | 8 (7-9) | NR | NR |
| Meinel, 2021 | VKA | 1486 | 81.3 ± 8.2 | 766/1,485 (52) | 1,486 (100) | NR | 156 (63.2) | 4 (2-11) | NR | 603.0 ± 1328.5 | NR |
|  | DOAC | 1,634 | 79.8 ± 8.9 | 883/1,633 (54) | 1,634 (100) | NR | 69 (14.9) | 6 (2-14) | NR | 697.8 ± 1451.0 | NR |
|  | Non-OAC | 5,059 | 79.4 ± 10.1 | 2,558/5,055 (51) | 5,059 (100) | NR | 1,544 (73.7) | 7 (3-15) | NR | 554.7 ± 1289.7 | NR |
| Pujara, 2024 | VKA | 15 | 74 (62-82) | 11 (73.3) | 10 (66.7) | ICA: 4 (26.7) MCA M1: 11 (73.3) MCA M2: 0 (0.0) | 0 (0.0) | 19 (12-24) | 4 (4-5) | NR | NR |
|  | DOAC | 14 | 69.5 (67-74) | 9 (64.3) | 9 (64.3) | ICA: 4 (28.6) MCA M1: 10 (71.4) MCA M2: 0 (0.0) | 3 (21.4) | 20 (18-21) | 4 (3-5) | NR | NR |
|  | Non-OAC | 151 | 65 (57-75) | 89 (58.9) | 28 (18.5) | ICA: 72 (47.7) MCA M1: 72 (47.7) MCA M2: 7 (4.6) | 34 (22.5) | 19 (15-23) | 4 (3-5) | NR | NR |
| Ramos-Araque, 2020 | DOAC | 81 | 76.37±9.79 | 43 (53) | 78 (96) | MCA-M1: 49 (61) Tandem: 6 (7) | NR | 15 (9-20) | 10 (8-10) | NR | NR |
|  | VKA | 193 | 76.66±10.2 | 92 (48) | 171 (89) | MCA-M1:118 (62) Tandem occlusion:14 (7) | NR | 18 (12-21) | 8 (7-10) | NR | NR |
|  | Non-OAC | 1,181 | 71.5±13.2 | 656 (55) | 153 (13) | MCA-M1 occlusion: 625 (54) Tandem occlusion: 230 (20) | NR | 16 (10-20) | 9 (8-10) | NR | NR |
| Rebello, 2015 | VKA | 29 | 68.7±13.57 | 12 (41) | 17 (58) | ACA: 2 (6.8) MCA M1: 19 (65.0) MCA M2: 4 (13.0) ICA-T: 5 (17.0) Vertebrobasilar: 0 (0.0) Tandem: 3 (10.0) | N/A | 19.3±5.5 | 8.2±1.8 | NR | NR |
|  | NOAC | 17 | 68.6±7.45 | 9 (52) | 14 (82) | ACA: 1 (5.8) MCA M1: 8 (47.0) MCA M2: 4 (23.5) ICA-T: 1 (5.8) Vertebrobasilar: 2 (11.7) Tandem: 3 (17) | N/A | 17.2±7.6 | 8.0±1.3 | NR | NR |
|  | Non-OAC | 265 | 64.0±14.7 | 138 (52) | 66 (24) | ACA: 7 (2.0) MCA M1: 128 (48.0) MCA M2: 30 (11.0) ICA-T: 50 (18.0) ET ICA: 42 (15.0) Vertebrobasilar: 42 (15.0) Tandem: 43 (16.0) | N/A | 18.3±6.3 | 7.6±1.4 | NR | NR |
| Uphaus, 2017 | VKA | 85 | NR | NR | NR | M1: 43 (52.4) M2: 3 (3.7) Carotid T occlusion: 13 (15.9) ICA: 3 (3.7) BA: 19 (23.2) Vertebral artery, V4: 0 (0.0) Other: 1 (1.2) | 22 (25.9) | NR | NR | NR | NR |
|  | Non-OAC | 730 | NR | NR | NR | M1: 361 (50.0) M2: 37 (5.1) Carotid T occlusion: 129 (17.9) ICA: 30 (4.2) BA: 118 (16.3) Vertebral artery, V4: 13 (1.8) Other: 34 (4.7) | 533 (73.1) | NR | NR | NR | NR |
| Wong, 2018 | VKA | 23 | 72.5 (59–78.5) | 19 (52.8) | 31 (86.1) | MCA (M1/M2): 26 (72.2) ICA: 4 (11.1) Tandem occlusion (ICA/MCA): 5 (13.9) BA: 1 (2.8) | 0 (0.0) | 16 (9.5-20.5) | NR | 280 (205.5–325) | NR |
|  | DOAC | 13 |  |  |  |  | 0 (0.0) |  | NR |  | NR |
|  | Non-OAC | 66 | 70.5 (57–76) | 39 (59.1) | 28 (42.4) | MCA (M1/M2): 30 (45.5) ICA: 8 (12.1) Tandem occlusion (ICA/MCA): 13 (19.70) BA: 15 (22.7) | 0 (0.0) | 16.5 (10-20) | NR | 346 (262–400) | NR |
| Zapata-Wainberg, 2018 | VKA | 104 | 72.73±9.23 | 50 (44.2) | 104 (92.0) | Left hemisphere: 44 (38.9) Right hemisphere: 64 (56.6) Vertebrobasilar: 5 (4.4) | 17 (15.0) | 16 (9) | 8 (3) | 305 (163.75) | NR |
|  | DOAC | 9 |  |  |  |  | 0 (0.0) |  |  |  | NR |
|  | Non-OAC | 389 | 65.87±13.23 | 206 (53) | 96 (24.7) | Left hemisphere: 162 (41.6) Right hemisphere: 181 (46.5) Vertebrobasilar: 46 (11.9) | 233 (59.8) | 17 (9) | 8 (2) | 330 (165) | NR |

**Abbreviations**: OAC, Oral Anticoagulant; VKA, Vitamin K Antagonist; DOAC, Direct Oral Anticoagulant; NOAC, Novel Oral Anticoagulant; NIHSS, National Institutes of Health Stroke Scal; ASPECT, Alberta Stroke Program Early CT Score; TICI, thrombolysis in Cerebral Infarction; ICA, Internal Carotid Artery; MCA, Middle Cerebral Artery; ACA, Anterior Cerebral Artery; PCA, Posterior Cerebral Artery; BA, Basilar Artery; NR, Not Reported; EC, Extracranial; IC, Intracranial; IV tPA, Intravenous Tissue Plasminogen Activator; mTICI, modified Thrombolysis in Cerebral Infarction.

Figure 3: Sensitivity Analysis of VKA vs. Non-OAC ICH


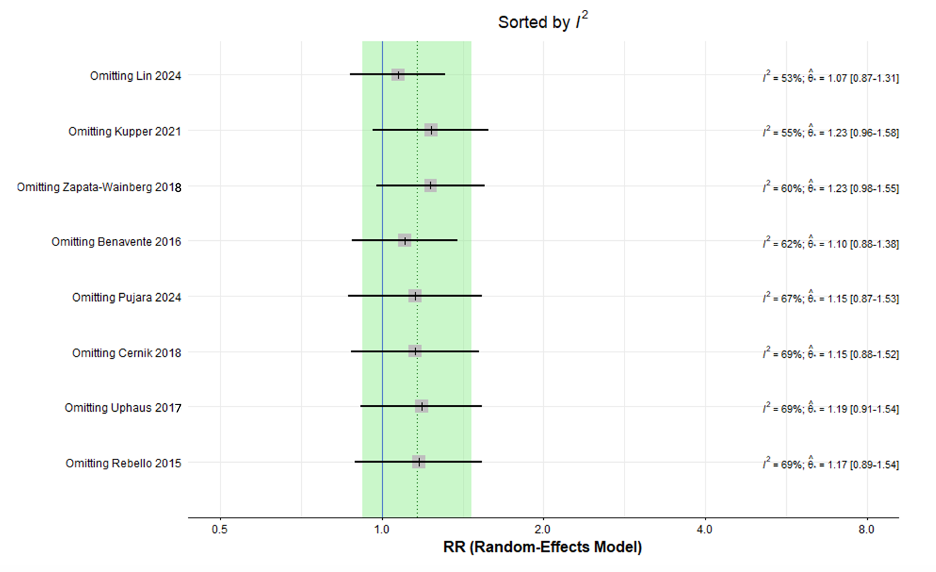


Figure 4: Sensitivity Analysis of Therapeutic DOAC vs. Non-OAC 90 days mRS 0-2


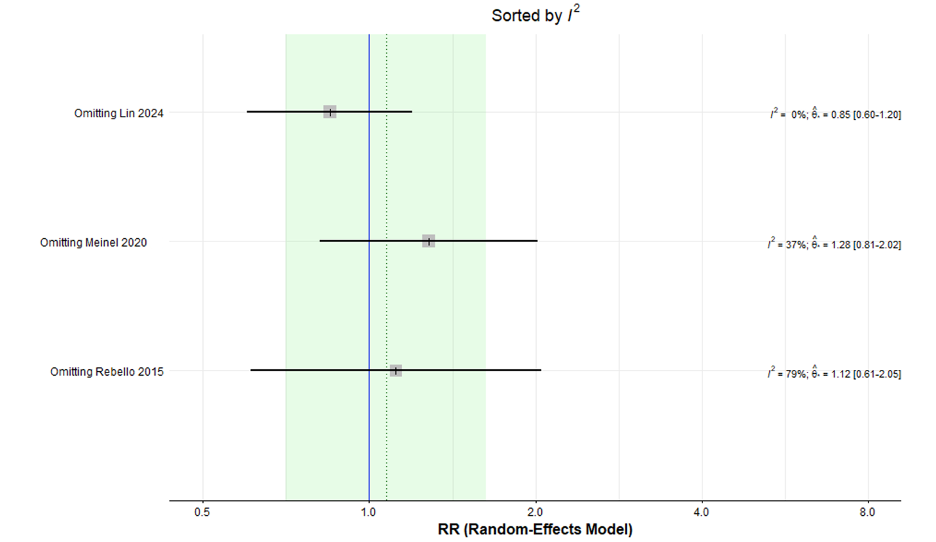


Figure 5: Sensitivity Analysis of DOAC vs. Non-OAC successful reperfusion


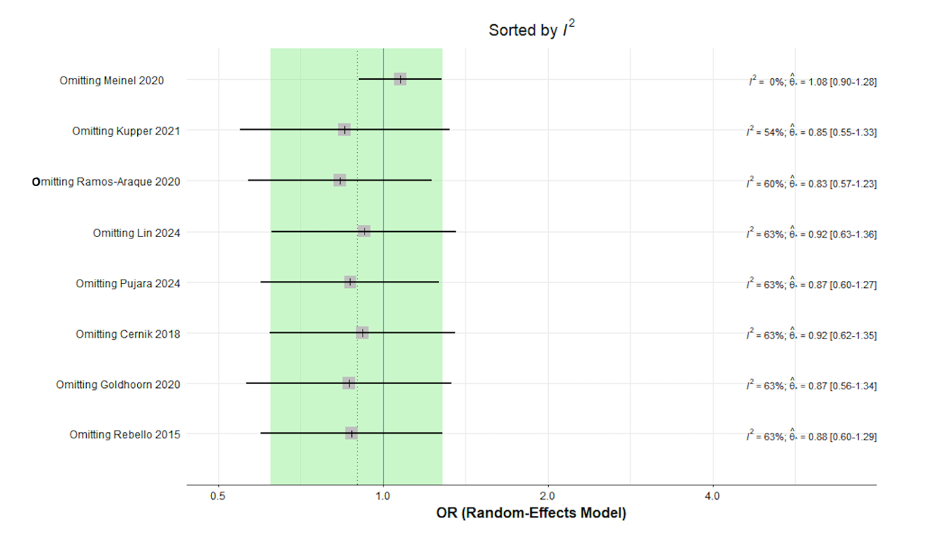


Table 3: Comparison of Meta-Analyses

| **Variables** | **Chen et al, 2022** | **Our study** |
| --- | --- | --- |
| Number of studies | 15 | 15 (5 new studies) |
| Sensitivity Analysis | NR | Yes |
| All patients underwent EVT | No | Yes |
| All patients compared to Non-OAC | No | Yes |
| All ICH | NR | ND |
| sICH | Favored DOAC | Favored DOAC |
| sICH therapeutic | ND | ND |
| Mortality | Favored DOAC | Favored DOAC |
| Mortality therapeutic | Favored DOAC | Favored DOAC |
| mRS 0-2 | ND | ND |
| mRS 0-2 therapeutic | Favored DOAC | Favored DOAC |
| Successful reperfusion | ND | ND |
| Successful reperfusion therapeutic | ND | ND |

**Abbreviations:** EVT, Endovascular Thrombectomy; Non-OAC: Non-Oral Anticoagulant; ICH, Intracranial Hemorrhage; sICH, symptomatic Intracranial Hemorrhage; mRS, modified Rankin Scale; ND, No Difference.
